# Thermal Resistance Enhancement and Wettability Amelioration of Poly(benzimidazole-aramid) Film by Silica Nanocomposites

**DOI:** 10.3390/polym16243563

**Published:** 2024-12-20

**Authors:** Jiabei Zhou, Xianzhu Zhong, Kenji Takada, Masayuki Yamaguchi, Tatsuo Kaneko

**Affiliations:** 1 Graduate School of Advanced Science and Technology, Japan Advanced Institute of Science and Technology (JAIST), 1-1 Asahidai, Nomi 923-1292, Japan; zhongxz@jaist.ac.jp (X.Z.); takadak@yz.yamagata-u.ac.jp (K.T.); m_yama@jaist.ac.jp (M.Y.); 2 Key Laboratory of Synthetic and Biological Colloids, School of Chemical and Material Engineering, Jiangnan University, 1800 Lihu Ave., Wuxi 214122, China; 3 Graduate School of Organic Materials Science, Yamagata University, 4-3-16, Jonan, Yonezawa 992-8510, Japan

**Keywords:** nanocomposite, polybenzimidazole, thermoresistance, dehydration, wettability amelioration

## Abstract

Polybenzimidazole (PBI) is a high-performance polymer known for its excellent thermal stability, mechanical strength, and chemical resistance, attributes that are derived from its unique structure comprising repeated benzene and imidazole rings. However, limitations such as relatively low thermal stability and moisture sensitivity restrict its application as a super engineering plastic. In this study, amide groups are incorporated into the PBI backbone to synthesize the copolymer poly(BI-*co*-A), effecting a structural modification at the molecular level. Additionally, silica nanospheres were composited into the poly(BI-*co*-A) film to further enhance its thermal performance. The resulting composite films exhibited remarkable thermal stability, with the temperature for 10% weight loss reaching as high as 761 °C. To address increased water absorption due to the high hydrophilicity of hydroxyl groups on the silica nanospheres’ surface, a dehydration treatment was applied in an electric furnace. This treatment produced a highly thermoresistant poly(BI-*co*-A) nanocomposite film with reduced wettability, making it suitable for applications in humid environments.

## 1. Introduction

Nanotechnology is expected to play a significant role in developing next-generation polymer materials and has attracted researchers’ attention in recent decades, including that of the nanoparticle composite method [1,2,3,4,5]. By incorporating nanoparticles such as carbon nanotubes, graphene, or ceramic fillers [6,7,8], numerous performances consisting of the thermal and mechanical properties of polymers could be enhanced without significantly increasing their weight. Generally, nanocomposites can enhance the heat dissipation properties of polymers, making them even more suitable for high-temperature applications [9,10,11,12]. Notably, nanoparticles can increase the strength and durability of polymers, making them resistant to wear and tear in extreme environments. Due to their high compatibility, using silica nanocomposites is a well-known strategy to combine with polymer matrices, leading to polymer materials with improved properties [13,14,15]. It is attributed that the unique synergy between silica and the polymer matrix gives the silica nanocomposite polymers a diverse set of properties, such as high mechanical strength and stiffness [16,17,18,19], thermal resistance and conductivity [20,21,22], optical transparency and diffusion [23,24,25,26], water and moisture resistance [27,28,29], and so on.

Herein, one king of super engineering plastics, polybenzimidazole (PBI), is known for its great potential as a thermal plastic owing to its unique structure, where the aromatic benzene rings and hetero rings endow PBI with high stability [30,31,32]. Simultaneously, the densely stacked aromatic rings resulted in a rigid backbone, bringing inferiority in overall flexibility, which limited the wide application of PBI [33,34]. To solve this problem, a modification to the backbone of PBI was made to decrease the aromaticity by introducing aliphatic amide groups to synthesize poly(benzimidazole-aramid) (poly(BI-*co*-A)) via the polymerization of bio-derivable monomer 3,4-diaminobenzoic acid (DABA), and 4-aminobenzoic acid (ABA), which could be produced by the fermentation of modified microorganisms with kraft pulp as the cellulosic resources [35,36]. This approach successfully improved the flexibility of PBI chains, and the processed pure poly(BI-*co*-A) film exhibited excellent thermal stability and mechanical properties. Despite improvements in thermomechanical properties achieved by introducing appropriate amounts of polyimide, significant enhancement in both mechanical properties and thermal stability remains challenging.

Due to the structural properties of the amide group, incorporating polyamide results in the formation of secondary amines, which have proton sites along the backbone, making it easy to form interchain hydrogen bonds. This feature makes poly(BI-*co*-A) an excellent material for silica composites, as the compatibility between poly(BI-*co*-A) and silica is significantly enhanced by hydrogen bonding. To explore improvements in thermomechanical performance, poly(BI-*co*-A) and silica nanocomposites were developed. With the inclusion of silica nanospheres, the thermal stability of poly(BI-*co*-A) was not only maintained but even enhanced at practical compositions. However, when combined with silica nanospheres, the poly(BI-*co*-A) film became more susceptible to water absorption due to the numerous hydrophilic hydroxyl groups on the silica surface. This water-absorption behavior notably impacted the thermomechanical properties, as the flexibility of the molecular chains and the toughness of the film were significantly altered when water penetrated the film’s texture and formed hydrogen bonds with the molecular chains. This effect poses challenges in applications where dimensional stability or mechanical strength is essential in humid environments.

To address this, a dehydration treatment of silica nanospheres, which could be achieved by thermal treatment at an extremely high temperature [37,38], was performed in an electric furnace to reduce surface wettability by decreasing hydrogen bonds within the poly(BI-*co*-A) nanocomposite films. In contrast to the general plastics with poor thermal stability, poly(BI-*co*-A) showed remarkable thermal resistance, making it incomparable suitability to this method. Through this approach, silica nanocomposite poly(BI-*co*-A) films with high thermal resistance and low wettability were produced, showing promise as engineering plastics in high-humidity environments.

## 2. Materials and Methods

### 2.1. Materials

3,4-diaminobenzoic acid (DABA, 99% purity) and 4-aminobenzoic acid (ABA, 99% purity) were purchased from Tokyo Chemical Industry Co., Ltd., Tokyo, Japan. Polyphosphoric acid (PPA, 80 wt% as P_2_O_5_) was purchased from FUJIFILM Wako Pure Chemical Corporation, Miyazaki, Japan. Silica nanospheres (seahostar@KE-P30, diameter: 300 nm) were purchased from Nippon Shokubai Co., Ltd., Osaka, Japan. Hydrofluoric acid (HF, 55% purity) was received from Morita Chemical Industries Co., Ltd., Osaka, Japan. Trifluoroacetic acid (TFA), Methanesulfonic acid (MSA), Sodium bicarbonate (NaHCO_3_), methanol, acetone, and other chemicals were purchased from Kanto Chemical Co., Inc., Tokyo, Japan. All the chemicals and reagents were used as received.

### 2.2. Measurements

Fourier transform infrared spectroscopy (FT-IR) was employed to characterize the fabricated poly(BI-*co*-A) films with various silica contents, using a Perkin-Elmer Spectrum with a diamond-attenuated total reflection (ATR) accessory, which was recorded in the wavenumber range of 4000 to 400 cm^−1^.

The size of silica nanospheres was revealed by scanning electron microscope (TM3030 plus tabletop SEM, Hitachi High-tech Corporation, Tokyo, Japan), which was also explored to investigate the surface and cross-sectional morphologies of the poly(BI-*co*-A) films. Energy-dispersive X-ray spectroscopy (EDS) was used to operate the elemental analysis of the poly(BI-*co*-A) films. All film specimens were pre-sputtered with Au powder under a vacuum for 30 s (thickness: 15 nm) (MSP-1S Magnetron Sputter, Vacuum Device Inc., Mito, Japan) before the SEM measurement.

Thermogravimetric analysis (TGA) (STA2700 system, Hitachi High-tech Corporation, Tokyo, Japan) was performed to investigate the thermal degradation of poly(BI-*co*-A) films. The sample films were placed in a platinum crucible heated to a maximum temperature of 800 °C at a heating rate of 10 °C min^−1^ under a nitrogen atmosphere.

The contact angle (CA) of the prepared poly(BI-*co*-A) films was measured by a contact angle meter (DM-501 series, Kyowa Interface Science Co., Ltd., Niiza, Japan) with a deionized water droplet of 1.5 μL at an ambient temperature.

### 2.3. Synthesis of Poly(benzimidazole-co-aramid)

**Scheme 1:**
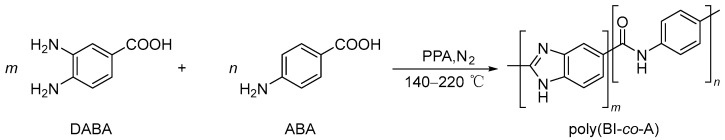
Synthesis of poly(BI-*co*-A) from DABA and ABA. (poly(BI-*co*-A = pol(benzimidazole-aramid); DABA = 3,4-diaminobenzoic acid; ABA = 4-aminobenzoic acid; PPA = poly(phosphoric acid)).

A typical synthetic procedure for poly(BI-*co*-A) was adopted for the polycondensation using high viscose poly(phosphoric acid) (PPA): 50 g PPA was added into a three-necked flask equipped with a magnetic stirrer and heated at 120 °C for 1 h in a nitrogen atmosphere. Subsequently, a composition of DABA (8.5 mmol) and ABA (1.5 mmol) was selected for copolymerization because the incorporation of a small amount of ABA unit into the benzimidazoles could enhance the thermal performance of the connected benzimidazole rings by strengthening their intermolecular interactions [39]. After stirring for 1 h to remove the moisture and dissolve the monomers completely, the solution was heated at 140 °C for 1 h, 160 °C for 1 h, and 180 °C for 8 h, respectively, until the solution became viscous and then maintained at 220 °C for another 12 h. During the process, the viscosity of the solution increased significantly with increasing polycondensation temperature, and the color of the solution changed from red to dark brown. The resulting viscous solution was precipitated into 500 mL of deionized water and washed for 12 h. After filtration, a brown solid was obtained and dried at 150 °C for 12 h. Finally, the solid was crushed into powder and neutralized with an aqueous solution of 5% NaHCO_3_, followed by repeated washing with deionized water until the pH of the cleaning liquid reached 7 (measured using pH test papers, Macherey-Nagel GmbH & Co. KG, Duren, Germany). The polymerization procedure is illustrated in Scheme 1.

### 2.4. Fabrication of Pure Poly(BI-co-A) Film

Due to the insolubility in normal organic solvents, a mixture solvent of TFA and MSA was prepared to dissolve fabricated pure poly(BI-*co*-A) film; poly(BI-*co*-A) powder (50 mg) and a mixture of TFA (3 mL) with 2 drops of MSA were merged into a screw-capped bottle equipped with a magnetic stirrer. After stirring at ambient temperature for 24 h until a transparent solution was formed, the poly(BI-*co*-A) solution was cast onto a silicon wafer and further dried to obtain a brown-colored film. Subsequently, the solvent-evaporated poly(BI-*co*-A) film was immersed in deionized water to remove any residue. After washing with deionized water repeatedly, the poly(BI-*co*-A) film was further dried at 200 °C for 12 h under vacuum.

### 2.5. Fabrication of Silica Nanocomposite Poly(BI-co-A) Films

**Figure 1:**
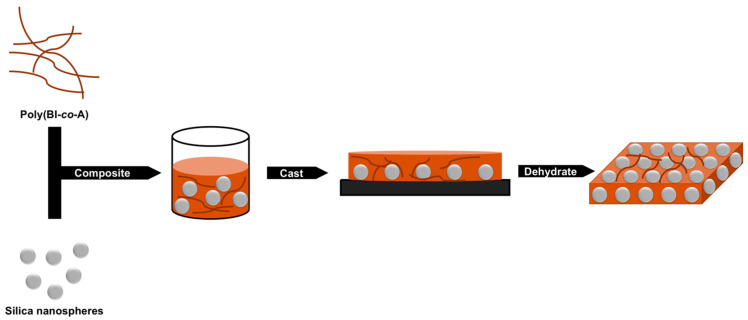
Schematic illustration of the fabrication process of silica nanocomposite poly(BI-*co*-A) films. (poly(BI-*co*-A) = poly(benzimidazole-aramid)).

Regarding the fabrication process of silica nanocomposite poly(BI-*co*-A) films (Figure 1), first, the poly(BI-*co*-A) powder (90, 80, 70, 60, and 50 mg) was dissolved in the mixture of the TFA and MSA solvent. On the other hand, silica nanospheres (10, 20, 30, 40, and 50 mg) with an average size of 300 nm were dispersed in TFA homogeneously by ultrasonication at ambient temperature for 1 h before compositing with the polymer solution. After the silica nanosphere dispersion was added into the completely dissolved poly(BI-*co*-A) solution, the mixing solution was further ultrasonicated for another 1 h before film casting. Poly(BI-*co*-A) films with various silica contents were fabricated using the two above-mentioned solutions with different weight percents of the mass of silica nanospheres to the total weight of poly(BI-*co*-A) and silica nanospheres (i.e., 10, 20, 30, 40, and 50%). As for the film casting process, the mixture solutions of silica nanocomposite poly(BI-*co*-A) solution were poured onto a silicon wafer substrate and then evaporated at room temperature. Afterward, the silica nanocomposite poly(BI-*co*-A) films were peeled off the silicon substrate and then repeatedly washed with deionized water. After the pH of the filtrate became 7, which was tested using pH test paper, the silica nanocomposite poly(BI-*co*-A) films were further dried at 200 °C in a vacuum oven. The prepared films above were named silica nanocomposite poly(BI-*co*-A)-X% films, where X corresponds to the composited content of silica nanospheres.

### 2.6. Dehydration Treatment

The obtained silica nanocomposite poly(BI-*co*-A) films were pre-heated at 100 °C for 12 h before the dehydration treatment. Subsequently, ceramic combustion boats were used to sandwich silica nanocomposite poly(BI-*co*-A) films and further heated in an electric furnace at 500 °C for 1 h with a nitrogen atmosphere to complete the dehydration treatment.

## 3. Results and Discussion

### 3.1. FT-IR Spectra

**Figure 2:**
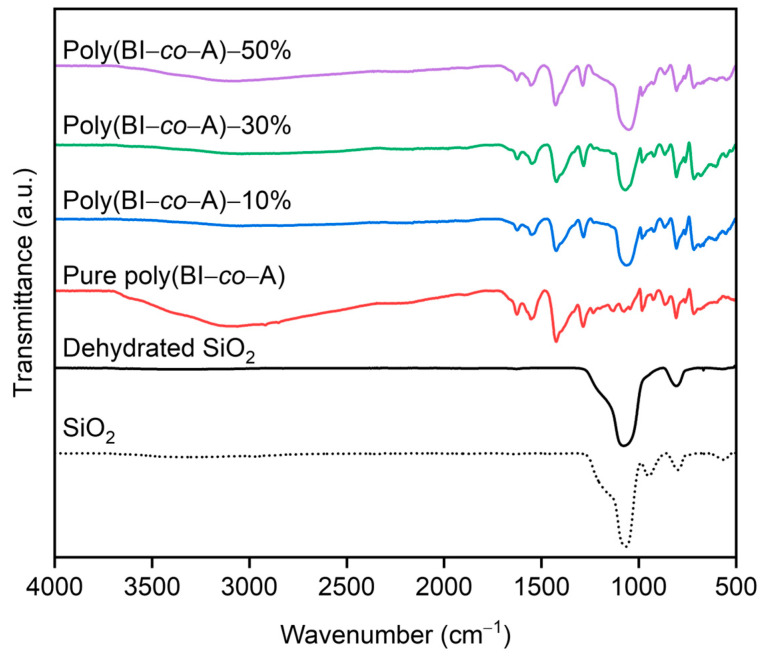
FT-IR spectra of bare and dehydrated silica nanospheres and poly(BI-*co*-A) films with various silica contents. (poly(BI-*co*-A) = poly(benzimidazole-aramid)).

Fourier transform infrared spectroscopy (FT-IR) was employed to identify the chemical structures of silica nanospheres, and pure and composited poly(BI-*co*-A) films (Figure 2). It is obvious that a broad absorption peak at about 1000–1270 cm^−1^ is assigned to the Si-O-Si asymmetric stretching vibrations, while a weak absorption peak at 794 cm^−1^ is assigned to the in-plane Si-O-Si deformation vibration. Moreover, the medium absorption peaks of the -OH group are characterized at about 3300 cm^−1^, and the characterized bending and stretching vibration of Si-OH is assigned at 950 cm^−1^ in the bare silica nanospheres [40]. Conversely, the disappearance of the Si-OH absorption peak in the dehydrated silica spectrum indicates the hydration of silanol functional groups on the surface of the silica nanospheres. Furthermore, a short peak shift in in-plane Si-O-Si deformation vibration from 794 to 804 cm^−1^ was also detected, implying a stronger combination bond [41], which suggests the successful hydration of silica nanospheres at 500 °C.

On the other hand, the chemical structure of the synthesized poly(BI-*co*-A), using high-viscose poly(phosphoric acid), was also confirmed. The characteristic peaks at 1747, 1625, 1550, and 1285 were assigned to the stretching vibrations of the C=O, C=N, C=C, and C-N groups, respectively, indicating the accomplished polycondensation of poly(BI-*co*-A). Additionally, a superb broad absorption peak at a high wavenumber region could be attributed to the numerous -NH and -OH groups from bonding and free water, implying the high hydrophilicity of pure poly(BI-*co*-A) film. Regarding the silica nanocomposite poly(BI-*co*-A) films, a broad absorption peak of Si-O-Si asymmetric stretching vibrations at about 1000 cm^−1^ and a weak absorption peak of the in-plane Si-O-Si deformation at about 800 cm^−1^ were observed in all the nanocomposite poly(BI-*co*-A) film spectra, demonstrating the successful composition of silica nanospheres into the poly(BI-*co*-A) film. Moreover, a remarkable reduction in the absorption peak of the -OH groups occurred in the poly(BI-*co*-A) film with silica nanospheres of 10 wt% content, indicating that the hydrophilicity decreased significantly after the dehydration treatment to the silica nanospheres. Although the strength of the absorption peak of the -OH group increased as increasing the silica nanospheres content from 10 to 50 wt%, which is derived from the uncompleted dehydration of silanol functional groups on the silica nanospheres, the poly(BI-*co*-A) film with 50 wt% silica nanospheres composition showed greater improved hydrophobicity than the pure poly(BI-*co*-A) film.

### 3.2. SEM Morphologies

**Figure 3:**
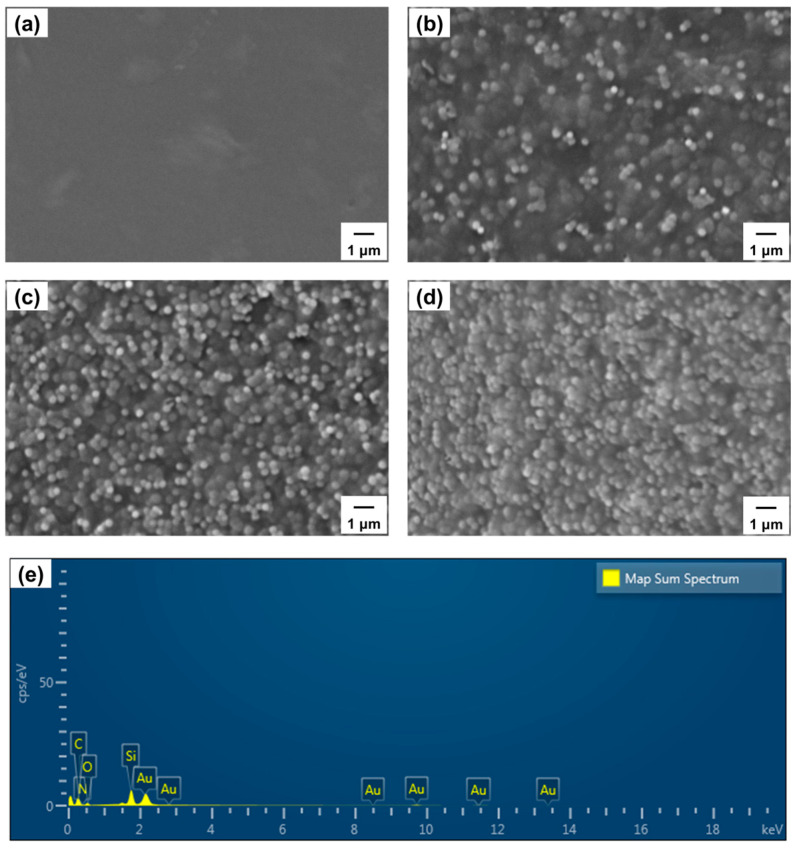
SEM images of pure (**a**) and silica nanocomposite poly(BI-*co*-A) films with silica contents of (**b**) 10%, (**c**) 30%, and (**d**) 50%, and (**e**) EDS elemental analysis of (**d**). (poly(BI-*co*-A) = poly(benzimidazole-aramid); SEM = scanning electron microscopy; EDS = energy-dispersive X-ray spectroscopy).

A scanning electron microscope (SEM) was used to investigate the morphology of the fabricated poly(BI-*co*-A) films with various silica nanosphere contents (Figure 3). According to the surface images, the original poly(BI-*co*-A) film exhibited a relatively uniform surface (Figure 3a), whereas all composite films had silica nanospheres on their surfaces, measuring approximately 300 nm in nano size. Additionally, the SEM images confirmed the increased silica content with an increase in the number of silica nanospheres (Figure 3b–d), indicating the successful preparation of silica composite poly(BI-*co*-A) films. The EDS mapping results verified the presence of silica nanospheres, with atomic percentages of 7.39 at% for silicon and 13.05 at% for silicon and oxygen, respectively (Table 1). Furthermore, no elemental sulfur or fluorine was detected, suggesting that the solvents used in the fabrication process were completely removed, which further demonstrates the effective integration of silica into the poly(BI-*co*-A) film.

### 3.3. TGA

**Figure 4:**
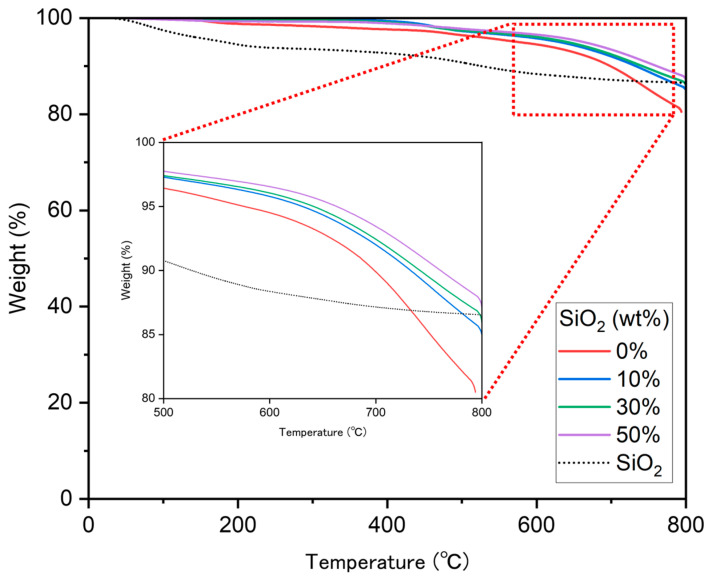
TGA curves of silica nanospheres and poly(BI-*co*-A) films with various silica contents (the inserted graph is the enlarged area at a high-temperature region). (TGA = thermogravimetric analysis; poly(BI-*co*-A) = poly(benzimidazole-aramid)).

**Figure 5:**
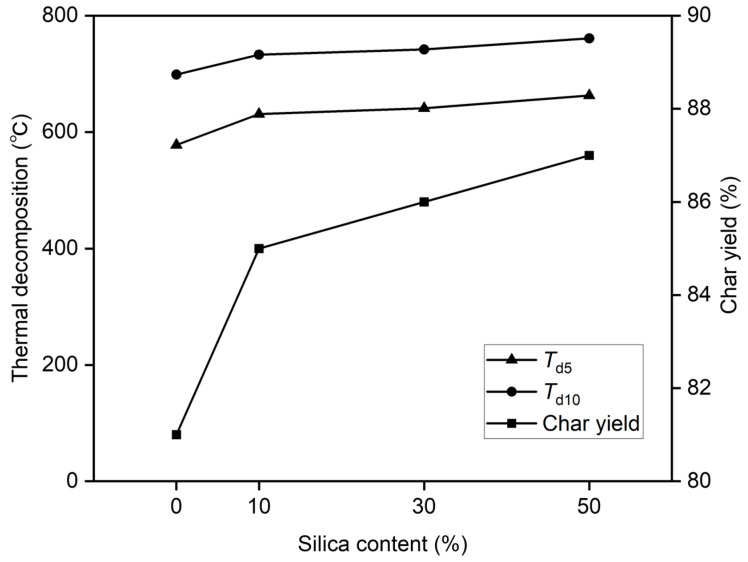
Thermal properties of poly(BI-*co*-A) films with various silica contents. (poly(BI-*co*-A) = poly(benzimidazole-aramid)).

Thermogravimetric analysis (TGA) was employed to assess the thermal properties of bare silica nanospheres, pure poly(BI-*co*-A) films, and their silica nanocomposite films (Figure 4). Despite the dehydration temperature, which is affected by the shape and size of silica nanoparticles [37,38], the thermal properties of the silica nanospheres used in this work were also investigated. A weight decrease was observed before approximately 220 °C could be attributed to the adsorbed water, while dehydration occurred at approximately 430 °C. Compared to pure poly(BI-*co*-A), poly(BI-*co*-A) silica nanocomposite films remain stable until approximately 400 °C, suggesting the high thermal stability of the poly(BI-*co*-A) silica nanocomposite films. Furthermore, 5% weight loss temperature (*T*_d5_) and 10% weight loss temperature (*T*_d10_) are taken as indices to evaluate the thermal stability (Figure 5). Poly (BI-*co*-A) silica nanocomposite films exhibit significantly higher *T*_d5_ and *T*_d10_ than pure poly(BI-*co*-A) films, and the thermal degradation temperatures increase along with the amount of the silica content. *T*_d5_ ranges from 631 °C to 663 °C as the silica composition increases from 10% to 50%. *T*_d10_ increases from 699 °C to 761 °C. The enhanced thermal properties could be attributed to the strengthened interfacial interactions between silica nanospheres and the polymer matrix, such as the fabricated hydrogen bonds from the residual hydroxy groups and the shielding effect of silica nanospheres [38]. Moreover, the hard and rigid bonds of Si-O-Si, showing an extremely high decomposition temperature, also contributed to improving the thermal properties of silica nanocomposite poly(BI-*co*-A) films. In addition, the char yield of the composites varies from 81% to 87% (Table 2); these thermal properties distinguish this series of composites in all the polymeric materials, implying their potential applications on thermal-retardant and non-flammable materials in extreme environments.

### 3.4. Contact Angle Test

**Figure 6:**
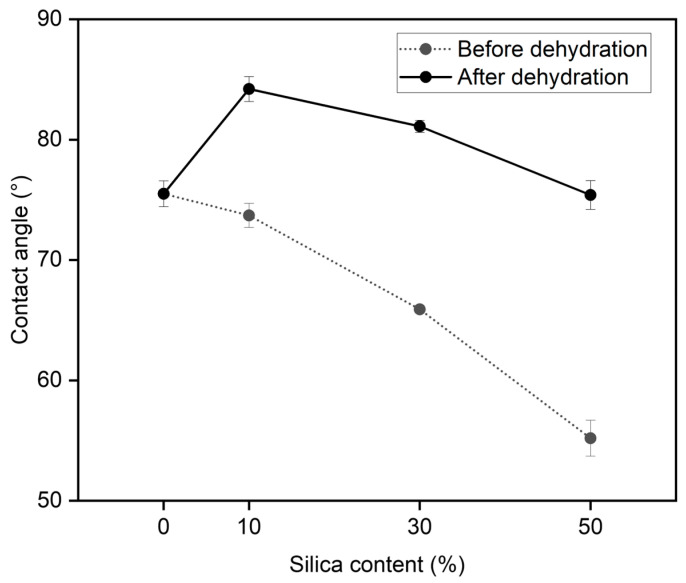
Surface wettability results of poly(BI-*co*-A) films with various silica contents.

A contact angle (CA) meter was used to assess the effect of silica nanosphere composition on the surface wettability of the fabricated poly(BI-*co*-A) films (Figure 6). The pure poly(BI-*co*-A) film exhibited a CA of 75.5°, indicating slight hydrophilicity. This is due to the weak interaction between water and the polymer film; although nitrogen and hydrogen atoms may form hydrogen bonds, most of these are interchain hydrogen bonds, limiting hydrogen bonding between water and the film surface. However, with increasing silica content, the CA showed a decreasing trend, with values of 73.7°, 65.9°, and 55.2° for 10%, 30%, and 50% silica composition, respectively, indicating that the incorporation of silica significantly increases the films’ wettability. This increase is attributed to hydroxyl functional groups on the silica nanospheres, which make the surface highly hydrophilic. The films’ wettability was reduced through dehydration treatment. After dehydration, the CA values increased to 84.2°, 81.1°, and 75.4° for 10%, 30%, and 50% silica content, respectively (Table 3), demonstrating that the wettability was reduced via decreasing the hydroxyl groups from the surface of the silica nanospheres by dehydration treatment. Nevertheless, the dehydration treatment in an electric furnace was proven to be a significant and simple method for improving the water resistance or wettability of polymer films, which could further expand their wide applications under humid and harsh environments as a super engineering nanocomposite plastic.

## 4. Conclusions

In conclusion, poly(BI-*co*-A) was successfully synthesized from bio-derivable monomers, DABA, and ABA, using high-viscosity poly(phosphoric acid), with its chemical structure confirmed via FT-IR. Due to its insolubility in common organic solvents, a mixture of TFA and MSA was used to process the poly(BI-*co*-A) film. Silica nanospheres were incorporated into poly(BI-*co*-A) to craft a silica nanocomposite. The obtained composite film was confirmed by FT-IR and SEM, which revealed a uniform dispersion of approximately 300 nm silica nanospheres. Poly(BI-*co*-A) nanocomposites exhibited a 5% degradation temperature increasing from 578 to 663 °C and a 10% degradation temperature rising from 699 to 761 °C. However, the nanocomposite films exhibited increased surface wettability as the contact angle decreased from 75.5° to 55.2° due to additional hydroxyl groups on the silica surface. To mitigate this, a dehydration treatment was applied in an electric furnace, increasing the contact angle to 84.2° by reducing hydrogen bonding in the film.

The fabricated silica nanocomposite poly(BI-*co*-A) films exhibited enhanced thermal resistance, comparable to some metals, suggesting their potential as a replacement for heavier metallic and inorganic materials. Furthermore, the optimized wettability of the poly(BI-*co*-A) films can enhance their suitability for extreme applications, including in fuel cell membranes, barriers, and anti-fouling films.

## Figures and Tables

**Table 1:** Elemental analysis results of the fabricated silica nanocomposite poly(BI-*co*-A) film.

Element	Line Type	Weight %	Atomic %
C	K series	44.24	68.20
N	K series	6.71	8.87
O	K series	11.28	13.05
Si	K series	11.21	7.39
Au	M series	26.56	2.50
Total		100.00	100.00

**Table 2:** Thermal properties of the fabricated poly(BI-*co*-A) films with various silica contents.

Films	*T*_d5_ (°C)	*T*_d10_ (°C)	Char Yield (%)
poly(BI-*co*-A)-0%	578	699	81
poly(BI-*co*-A)-10%	631	733	85
poly(BI-*co*-A)-30%	641	742	86
poly(BI-*co*-A)-50%	663	761	87

Poly(BI-*co*-A) = poly(benzimidazole-aramid).

**Table 3:** Contact angle of pure poly(BI-*co*-A) films, before and after the dehydration of silica nanocomposite poly(BI-*co*-A) films with various silica contents.

Films	Contact Angle (°)	
poly(BI-*co*-A)-0%	75.5 ± 1.1	
	Before dehydration	After dehydration
poly(BI-*co*-A)-10%	73.7 ± 1.0	84.2 ± 1.0
poly(BI-*co*-A)-30%	65.9 ± 0.3	81.1 ± 0.5
poly(BI-*co*-A)-50%	55.2 ± 1.5	75.4 ± 1.2

## Data Availability

The raw data supporting the conclusions of this article will be made available by the authors upon request.
